# Supplementary material for: Content-rich biological network constructed by mining PubMed abstracts
Source: BMC Bioinformatics. 2004 Oct 8;5:147. doi: 10.1186/1471-2105-5-147 (PMC528731; doi:10.1186/1471-2105-5-147)
Supplement: Additional File 2 — The original results of the above study (non-essential files are deleted to keep the file size under the limit set by BMC bioinformatics). [file 1471-2105-5-147-S2.bz2 › chilibotAdditionalFile2/dip05/608ID1701053E10990/html/CSF3R_CSF3.html]

 


 **CSF3R** and **CSF3** 
  
Found 215 abstracts in PubMed, retrieved 05.  
 

 What does Google say? 
 PDF only 
| .edu only 

---

**Interactive relationship** (e.g. stimulation, inhibition, etc)

**Neutral relationship**- G CSF  [ **CSF3** ]  activates a receptor of the hematopoietin receptor superfamily, the G CSF  [ **CSF3** ]  receptor G CSF  [ **CSF3** ]  R  [ **CSF3R** ] , which subsequently triggers multiple signaling mechanisms.  Ref: 12687405 Rev Physiol Biochem Pharmacol< MedlineTA>Rev Physiol Biochem Pharmacol,

**Non-interactive relationship** (e.g. studied together, co-existance, homology, etc.)

- In approximately 20% of SCN cases, a truncation mutation is found in the cytoplasmic region of the granulocyte colony stimulating factor  [ **CSF3** ]  receptor  [ **CSF3R** ]  G CSFR.  Ref: 12672695 Blood, 2003
- Here we review how these mechanisms contribute to the specific responses of hematopoietic cells to G CSF  [ **CSF3** ]  and how perturbations in the function of the G CSF  [ **CSF3** ]  R  [ **CSF3R** ]  are implicated in various types of myeloid disease.  Ref: 12687405 Rev Physiol Biochem Pharmacol< MedlineTA>Rev Physiol Biochem Pharmacol, 2003
- Real time PCR analysis revealed that TPT augmented the expression not only of CD18 but also of components of superoxide generating NADPH oxidase, p47phox, 2.7 fold, and p67phox, 2.0 fold, and of granulocyte colony stimulating factor  [ **CSF3** ]  receptor  [ **CSF3R** ]  G CSFR, 3.0 fold.  Ref: 12788061 Biochem Biophys Res Commun, 2003
- Bone marrow derived primitive CD34 and CD33 CD34 progenitor cells from SCN patients evolving to AML, all with mutations in the granulocyte colony stimulating factor  [ **CSF3** ]  receptor  [ **CSF3R** ]  G CSFR gene, demonstrated normal cell survival.  Ref: 12763135 Exp Hematol, 2003
- IL 10 Milenia Biotec, Bad Nauheim, Germany, gammaINF Diaclone, Besancon, France and G CSF  [ **CSF3** ]  R  [ **CSF3R** ]  &.  Ref: 12788309 Cytokine, 2003
